# Secondary Structural Model of MALAT1 Becomes Unstructured in Chronic Myeloid Leukemia and Undergoes Structural Rearrangement in Cervical Cancer

**DOI:** 10.3390/ncrna7010006

**Published:** 2021-01-13

**Authors:** Matthew C. Wang, Phillip J. McCown, Grace E. Schiefelbein, Jessica A. Brown

**Affiliations:** Department of Chemistry and Biochemistry, University of Notre Dame, Notre Dame, IN 46556, USA; mwang7@alumni.nd.edu (M.C.W.); pjlmac@gmail.com (P.J.M.); gschiefe@nd.edu (G.E.S.)

**Keywords:** cancer, RNA secondary structure, DMS-Seq, PARIS, miRNAs

## Abstract

Long noncoding RNAs (lncRNAs) influence cellular function through binding events that often depend on the lncRNA secondary structure. One such lncRNA, metastasis-associated lung adenocarcinoma transcript 1 (MALAT1), is upregulated in many cancer types and has a myriad of protein- and miRNA-binding sites. Recently, a secondary structural model of MALAT1 in noncancerous cells was proposed to form 194 hairpins and 13 pseudoknots. That study postulated that, in cancer cells, the MALAT1 structure likely varies, thereby influencing cancer progression. This work analyzes how that structural model is expected to change in K562 cells, which originated from a patient with chronic myeloid leukemia (CML), and in HeLa cells, which originated from a patient with cervical cancer. Dimethyl sulfate-sequencing (DMS-Seq) data from K562 cells and psoralen analysis of RNA interactions and structure (PARIS) data from HeLa cells were compared to the working structural model of MALAT1 in noncancerous cells to identify sites that likely undergo structural alterations. MALAT1 in K562 cells is predicted to become more unstructured, with almost 60% of examined hairpins in noncancerous cells losing at least half of their base pairings. Conversely, MALAT1 in HeLa cells is predicted to largely maintain its structure, undergoing 18 novel structural rearrangements. Moreover, 50 validated miRNA-binding sites are affected by putative secondary structural changes in both cancer types, such as miR-217 in K562 cells and miR-20a in HeLa cells. Structural changes unique to K562 cells and HeLa cells provide new mechanistic leads into how the structure of MALAT1 may mediate cancer in a cell-type specific manner.

## 1. Introduction

Long noncoding RNAs (lncRNAs; all abbreviations henceforth can be found in Supplemental Table S1, “Abbreviations” tab) are involved in a wide array of biological functions in humans, including imprinting, cell differentiation, and disease proliferation [1,2]. lncRNAs regulate gene expression through multiple mechanisms, including alternative splicing [3], binding transcription factors [4,5], and binding microRNAs (miRNAs) [6]. Specifically, lncRNAs can act as competing endogenous RNAs (ceRNAs) and sponge miRNAs, thus hindering those miRNAs from promoting degradation of their intended mRNA targets [7,8,9,10]. In some situations, miRNA binding can even promote lncRNA degradation [9]. The ability of lncRNAs to interact with other RNAs and proteins is largely mediated by secondary structure. For instance, lncRNAs can sponge miRNAs when the binding site is free of secondary structures (e.g., hairpins and pseudoknots) and/or proteins. In cancer and other human diseases, the proper functioning of lncRNAs is directly impacted by miRNA-binding events, where miRNA sponging can promote mRNA dysregulation and aberrant gene expression [11]. lncRNAs are often associated with oncogenic or tumor-suppressing effects and have high potential for use as cancer biomarkers [12]. While the structure dictates function of RNA and proteins, the distinct functional roles of structural elements in many lncRNAs are unclear.

One notable cancer-associated lncRNA is human metastasis-associated lung adenocarcinoma transcript 1 (MALAT1), whose longest isoform has over 8700 nucleotides [13] and its mature form terminates in a triple helix at its 3′ end [14]. Additionally, MALAT1 has hnRNPC- and hnRNPG-binding sites which are made available when *N*^6^-methyladenosine (m^6^A) modifications destabilize characterized hairpins [15,16,17]. Numerous roles for MALAT1 have been proposed, including regulation of pre-mRNA splicing, transcriptional regulation through binding of transcription factors, and acting as a ceRNA [18]. MALAT1 has previously been linked to SR protein phosphorylation and alternative splicing of pre-mRNAs [3]. Aberrant MALAT1 upregulation is considered oncogenic and occurs in breast, cervical, liver, and lung cancers, among others [18]. Moreover, MALAT1 has numerous known miRNA- and protein-binding partners, some of which mediate cancer. For example, the sponging of miR-217 by MALAT1 promotes dasatinib resistance in K562 cells by upregulating AGR2 [19]. Similarly, MALAT1 sponges the tumor suppressors miR-145 [20] and miR-202-3p [21], prompting invasiveness and epithelial-mesenchymal transitioning in HeLa cells [22]. The strong correlation between MALAT1 expression and cancer [18], as well as numerous known interactions with miRNAs and proteins that can bind to MALAT1, has made MALAT1 a promising biomarker and anticancer therapeutic target [23]. While increased expression of MALAT1 has been correlated with cancer or severe cancer phenotypes, how the roles of MALAT1 are influenced by its myriad structural elements is not yet clear.

Previously, a secondary structural model of human MALAT1 in noncancerous cells was proposed [15]. The model posits 194 helices and 13 pseudoknots covering 8425 nucleotides of MALAT1 and identified several unique and dynamic MALAT1 secondary structures, including a putative m^6^A switch that regulates miRNA binding [15]. According to this model, when there is a lack of methylation at A5044 in HeLa cells, a short hairpin is favored over a pseudoknot, thereby possibly increasing the accessibility of cancer-associated miR-101-3p, miR-217-5p, and miR-383-5p to their binding sites in MALAT1 [15,24,25,26]. Based on this structural switch and other cell-dependent structural predictions, we decided to examine structural data for MALAT1 in cancerous contexts. Herein, the working noncancer-derived secondary structural model of MALAT1 was compared to previously published RNA structural probing data in chronic myeloid leukemia (CML)-derived K562 cells [27] and in cervical cancer-derived HeLa cells [28,29] to identify putative differences between cancerous and noncancerous MALAT1 structural models [15]. DMS-Seq data from K562 cells [27] and PARIS data from HeLa cells [28,29] were used to determine how the structural model of MALAT1 changes in cancer. Both datasets were analyzed to generate a more complete picture of MALAT1, as the output for DMS-Seq provides structural information for only adenosine and cytidine and PARIS detects only psoralen-crosslinked duplexes [27,28,29]. From these comparative analyses, MALAT1 is predicted to become unstructured in K562 cells, with 59 of 101 analyzed hairpins losing at least half of the base pairs predicted in MALAT1 in a noncancerous context. Conversely, the MALAT1 model is largely maintained in HeLa cells, but 18 novel RNA-RNA interactions suggest structural rearrangements predominate. These structural changes would subsequently impact accessibility of 50 miRNA-binding sites. For instance, the binding site for miR-217, which is associated with tumor suppression [30], is subject to linearization in K562 cells, which would allow MALAT1 to sponge miR-217. Overall, this work highlights the potential, novel dynamic secondary structural changes in K562 and HeLa cells, whereby differential effects on miRNA-binding sites, protein-binding sites, RNA modifications, single nucleotide polymorphisms (SNPs), and cancer-associated mutations suggest mechanisms by which MALAT1 plays different roles in K562 and HeLa cells.

## 2. Results and Discussion

### 2.1. DMS-Seq Data Suggest Unfolding of MALAT1 Structure in K562 Cells

**Figure 1:**
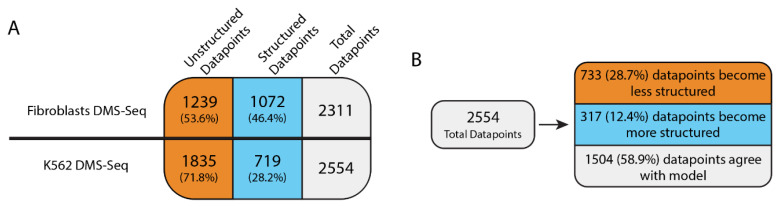
Summary of DMS-Seq data analysis. (**A**) 2311 available DMS-Seq datapoints (i.e., number of DMS-Seq reads corresponding to a single adenosine or cytidine) in human foreskin fibroblasts and 2554 DMS-Seq datapoints in K562 cells were classified as unstructured or structured based on whether they exceeded the 20-read or 250-read threshold, respectively (see Section 3 and [15]). (**B**) K562 DMS-Seq datapoints were compared to the working MALAT1 consensus model and further classified as losing structure, gaining structure, or agreeing with the model [15].

DMS-Seq involves chemically labeling RNA with DMS on unstructured adenosine and cytidine residues, which stops reverse transcriptase in a manner that can be detected by sequencing [27]. K562 DMS-Seq data [27] for MALAT1 (herein referred to as K562-MALAT1) were first analyzed to determine which adenosine and cytidine nucleotides are unstructured or structured (Supplemental Table S1). Of the 8425 nucleotides within the human MALAT1 transcript that were examined, 3951 nucleotides are either adenosine or cytidine. Of these, DMS-Seq data determined using MALAT1 isolated from K562 cells were available for 2554 adenosine and cytidine nucleotides, whereby 1835 DMS-Seq (71.8%) datapoints (i.e., number of DMS-Seq reads corresponding to a single adenosine or cytidine) were classified as unstructured and 719 DMS-Seq datapoints (28.2%) were classified as structured pursuant to the 250-read threshold (see Section 3 and Figure 1A). When K562-MALAT1 was compared to the working noncancerous MALAT1 model (Supplemental Table S1), 1504 datapoints (58.9%) agreed with the consensus model while 733 datapoints (28.7%) corresponded to loss of structure and 317 datapoints (12.4%) corresponded to gain of structure (Figure 1B). While the majority of K562-MALAT1 agreed with the MALAT1 consensus model, 41.1% of datapoints diverged from the noncancerous model, suggesting that wide-sweeping changes in MALAT1 secondary structure may occur in K562 cells.

**Figure 2:**
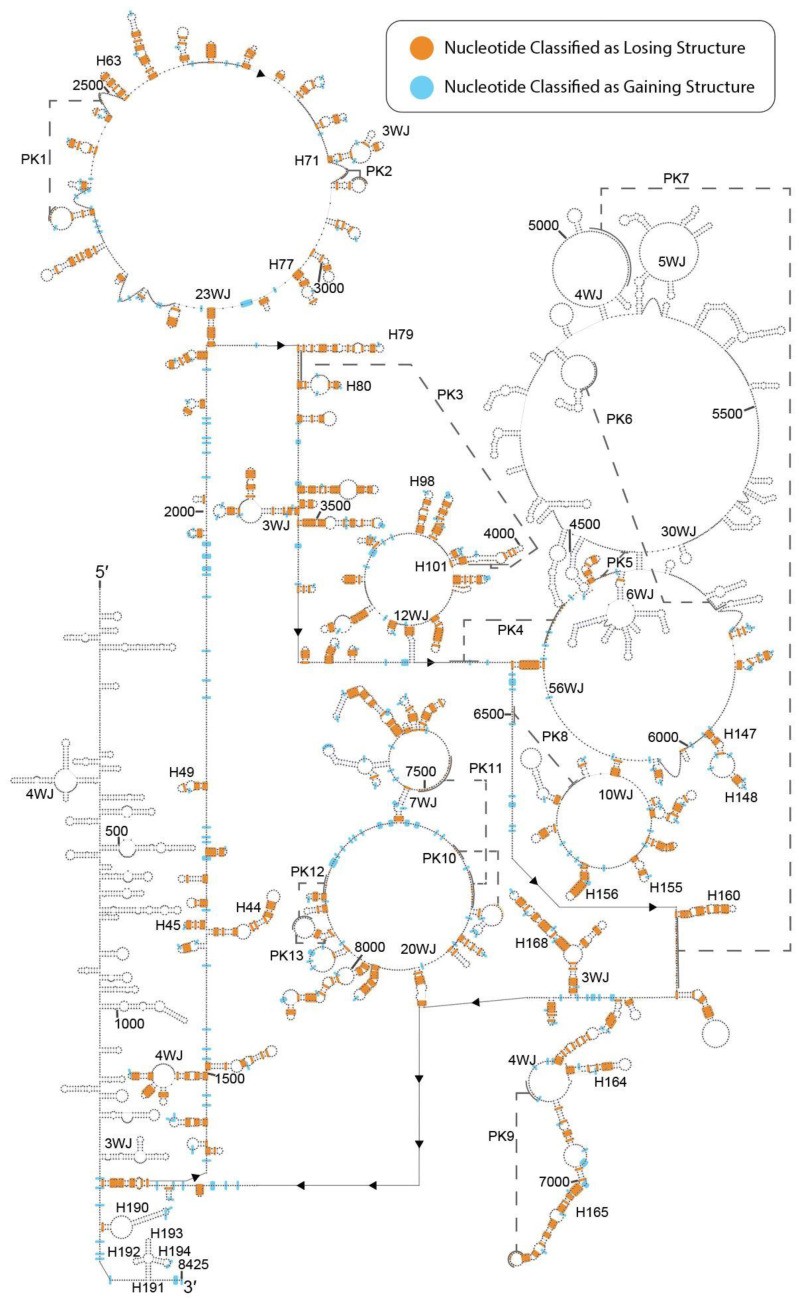
Map of K562 DMS-Seq structural changes onto the MALAT1 secondary structural model. K562 DMS-Seq data were available for nts 1284–4257 and nts 5839–8425. Of those indicating change in structure relative to the noncancerous consensus model, adenosine and cytidine residues that are predicted to lose structure are depicted in orange (733 datapoints) and those residues that are predicted to gain structure are depicted in blue (317 datapoints). Orange marks in hairpins have been extended to cover both nucleotides in a given base pair, and base pairs marked as unstructured have the two nucleotides moved apart to emphasize loss of structure. Labels for secondary structures correspond to those established for the working MALAT1 model in noncancerous cells [15]. Please note that PARIS-derived hairpins appear in the consensus model, although these hairpins were not considered in the differential structural analysis of K562-MALAT1.

**Figure 3:**
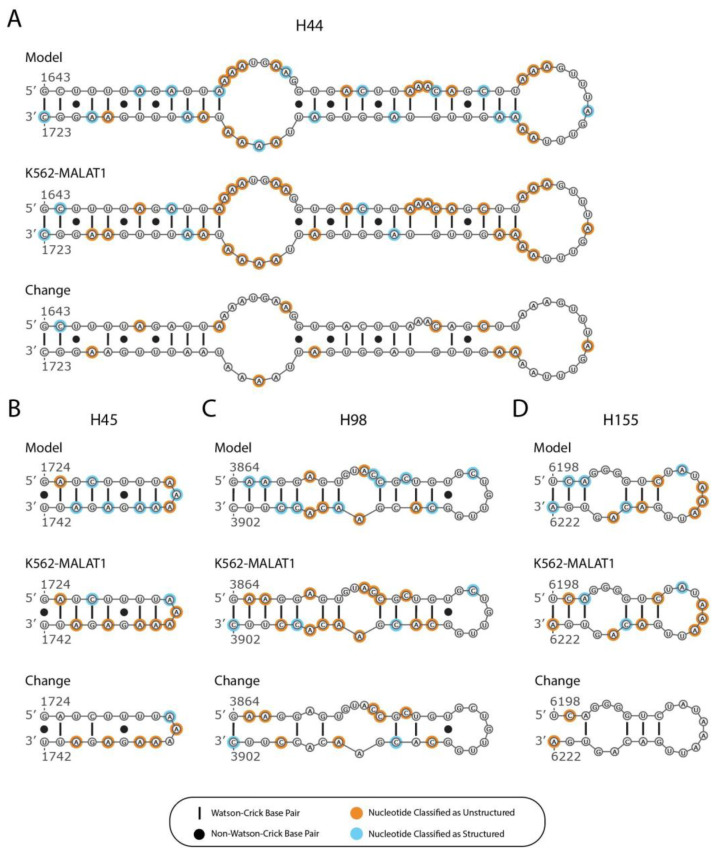
Hairpins predicted to be disrupted in K562-MALAT1. Loss of structure is shown for four hairpins: (**A**) H44, (**B**) H45, (**C**) H98, and (**D**) H155. Canonical base pairs are shown with a line and noncanonical base pairs are shown with a dot. Nucleotides with an orange background are classified as unstructured and nucleotides with a blue background are classified as structured. For each hairpin, the following schematics are shown from top to bottom: original secondary structure as predicted in the working MALAT1 model (“Model”) using fibroblast DMS-Seq data, the K562 DMS-Seq data (“K562-MALAT1”), and the K562 DMS-Seq datapoints that differ from their noncancerous counterparts (“Change”).

K562-MALAT1 data that corresponded to the loss or gain of structure were mapped onto the noncancerous MALAT1 model to examine how the secondary structure of MALAT1 may vary in K562 cells (Figure 2 and Supplemental Figure S1). Of the 194 hairpins predicted in the noncancerous MALAT1 model, 101 hairpins from the noncancerous model are supported by DMS-Seq data and 59 of these hairpins (58.4%) lose at least half of their base pairs in K562 cells. Among these hairpins are: H44, losing 13 of 25 base pairs (52.0%); H45, losing 5 of 8 base pairs (62.5%); H98, losing 8 of 12 base pairs (66.7%); and H155, losing 4 of 6 base pairs (66.7%) (Figure 3, “K562-MALAT1”). These correspond to novel losses of eight base pairs in H44, four base pairs in H45, six base pairs in H98, and two base pairs in H155 (Figure 3, “Change”). The comparative analysis suggests widespread loss of structural features within the context of K562-MALAT1. While individual nucleotides occasionally appear to gain structure in K562-MALAT1, the sporadic occurrences do not suggest the development of any unambiguous novel secondary structures in MALAT1 in K562 cells (Figure 2). It is worth noting that refolding of the MALAT1 structure based on the K562 DMS-Seq data is expected to produce a novel secondary structure of K562-MALAT1. However, DMS-Seq is the only major RNA structural probing dataset available for K562 cells and it lacks data for about 1600 nts from a central region of MALAT1; therefore, a novel model cannot be constructed and the analysis herein is restricted to identifying regions of MALAT1 that potentially change in K562 cells. Overall, K562-MALAT1 results suggest many hairpins in the working MALAT1 model lose structure, and unstructured regions remain unstructured. This result is in agreement with prior work using DMS-Seq data, which found loss of structure in mRNAs [27]. Cumulatively, the K562-MALAT1 data indicate general loss of structure in MALAT1, thereby suggesting possible functional ramifications within the context of K562 cells.

### 2.2. Predicted Secondary Structural Changes in K562-MALAT1 Would Impact Multiple RNA- and Protein-Binding Sites

Loss of secondary structure in K562-MALAT1 may signal that certain RNA- and protein-binding sites are now available in MALAT1, especially for single-stranded RNA-binding proteins. As such, this possibility was examined to identify the aberrant binding events in K562-MALAT1 that are different from binding and interaction events for MALAT1 in noncancerous conditions. miRNAs, ncRNAs, proteins, RNA modifications, SNPs, and cancer-associated mutations were re-aligned to MALAT1 to identify structure-function relationships that provide a starting point to examine their possible roles in CML (Supplemental Table S1).

**Figure 4:**
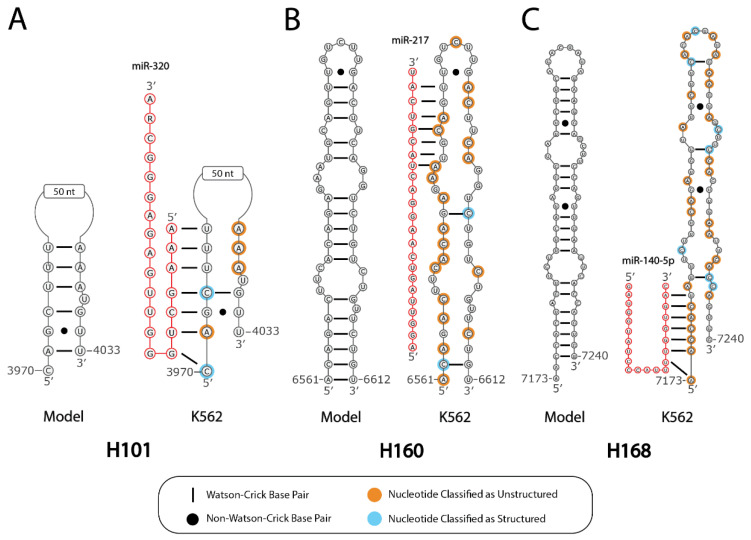
Structural loss in K562-MALAT1 improves accessibility of miRNA-binding sites. Three hairpin-miRNA interactions are shown: (**A**) miR-320 seed binding to H101, (**B**) miR-217 seed binding to H160, and (**C**) miR-140-5p seed binding to H168. Depicted is the predicted structure of each hairpin in noncancerous cells (“Model”) adjacent to the hairpin structure in K562-MALAT1 (“K562”), where the relevant base pairs are removed according to our analysis of DMS-Seq data. Nucleotides with an orange background are classified as unstructured and nucleotides with a blue background are classified as structured. The binding of a given miRNA (red) seed region to the K562-MALAT1 hairpin is shown. The nucleotide R denotes a G and A residue in miR-320a and miR-320b, respectively.

miRNAs are currently known to play a pivotal role in the development and progression of CML [31]. Of the 98 validated miRNA-binding sites in MALAT1, 28 sites occur in hairpins predicted to lose structure in K562-MALAT1, thereby increasing accessibility of binding site and potential for sponging (Supplemental Table S1). Examples include miR-320, which overlaps with H101; miR-217, which overlaps with H160; and miR-140-5p, which overlaps with H168 (Figure 4). miR-320 is considered a tumor suppressor in K562 cells, but K562 cells often bypass its action by transporting miR-320 to exosomes via hnRNPA1 [32]. Sponging of miR-320 by MALAT1 in K562 cells could also dampen the tumor-suppressive effects of miR-320, as is the case with lncRNA SNHG12 sponging miR-320 in gastric cancer [33]. miR-217 reportedly targets the mRNA of oncogenic protein AGR2 in K562 cells [19]. As decreases in unbound miR-217 accompany AGR2 upregulation and subsequent dasatinib resistance in K562 cells [19], sponging of miR-217 by MALAT1 may have similar effects in K562 cells. miR-140-5p has been linked to CML cell apoptosis via targeting of the SIX1 mRNA transcript [34]; therefore, possible sponging of miR-140-5p by MALAT1 in K562 cells may promote cell survival. These examples highlight how the novel availability of miRNA-binding sites in MALAT1 may aid in K562 cell progression via multiple miRNA-mediated mechanisms.

Eleven hairpins expected to lose structure in K562-MALAT1 (H49, H77, H79, H80, H147, H148, H155, H156, H164, H165, H168) overlap with eight of the ten U1 snRNA-binding sites (nts 1825–1925, 3015–3067, 3152–3185, 5924–6023, 6127–6277, 6850–6884, 6985–7045, and 7138–7206) (Supplemental Table S1). U1 snRNA is known to be mutated in multiple cancer types to promote aberrant gene splicing patterns [35]. Although the roles of U1-MALAT1 interactions have not been elucidated, it is conceivable that binding of U1 snRNA to MALAT1 may also contribute toward alternate, oncogenic splicing patterns that promote CML. Furthermore, one U1 snRNA-binding site (nts 3152–3185) overlaps with a HuR/ELAV1-binding site (nts 3158–3163) that may become accessible upon loss of H79 in K562-MALAT1. Although the effects of HuR-MALAT1 binding on CML have not been investigated, the HuR-MALAT1 RNP complex has been shown to stop breast cancer cells from undergoing epithelial-mesenchymal transitioning by decreasing the levels of CD133 [36]. Thus, increased HuR-MALAT1 binding in K562 cells is expected to hinder cancer progression, suggesting the existence of alternate pathways by which HuR-MALAT1 binding affects K562 cells. HuR typically binds to mRNAs in cancer in order to promote cancerous functions, such as metastasis and apoptosis resistance [37]. Thus, competition between HuR and U1 snRNA for a binding site around nt 3160 may point to a carefully controlled cancer-promoting mechanism mediated by MALAT1. In general, characterized protein-binding sites on MALAT1 are expected to become more available as a result of widespread structural loss and these changes in protein-MALAT1 binding predicted by K562 DMS-Seq data hint at novel pathways to explore further.

Besides RNA- and protein-binding sites, RNA structure can also be modulated by RNA modifications, SNPs, and cancer-associated mutations. RNA modifications on MALAT1 in K562 cells are undetermined, so modification sites from other cell lines were used. Of all the 82 m^6^A modifications mapped to MALAT1 at single-nucleotide resolution in HEK293, HEK293T, and HeLa cells [24,38,39,40,41], 61 m^6^A modifications either overlap with hairpins predicted to lose structure or, if not overlapping with hairpins, correspond to adenosines predicted to be unstructured (Supplemental Table S1). The METTL3/14 complex, which is responsible for about 80% of m^6^A marks in human mRNAs and ncRNAs, has no strong preference for ssRNA or dsRNA [42], suggesting secondary structural changes are insufficient to predict changes in m^6^A levels caused by METTL3/14. It is worth noting that the METTL3/14 complex is considered tumor suppressive and is downregulated in cancers like endometrial cancer, whereas m^6^A erasers like ALKBH5 and FTO are oncogenic and often upregulated in cancers like acute myeloid leukemia (AML) and breast cancer [43]. Correspondingly, ALKBH5 is associated with MALAT1 upregulation [44] and FTO regulates MALAT1 levels via demethylation [45]. ALKBH5 does not discriminate between ssRNA and dsRNA [46] and FTO targets ssRNA [47]; therefore, m^6^A marks in MALAT1 are potential substrates for both m^6^A erasers. Also notable, m^6^A2515 enables the binding of hnRNPG to MALAT1 at H63 [16], which is lost in K562-MALAT1 (Figure 2). As hnRNPG has stronger binding affinity for ssRNA, particularly in A-rich regions, [48,49] and has generally been associated with tumor suppressive effects [50,51,52], increased MALAT1-hnRNPG binding may decrease the tumor suppressive activity of hnRNPG and may promote K562 cell progression. While the functional effects of any aberrant methylation patterns are difficult to predict in CML, m^6^A modifications and their roles in RNA regulation and function, particularly with regard to mRNAs where such modifications are the most common, have been explored in attempts to develop novel cancer biomarkers and treatments [53]. Therefore, understanding how structural alterations in MALAT1 modulate m^6^A modification sites in K562 cells is of particular interest.

**Figure 5:**
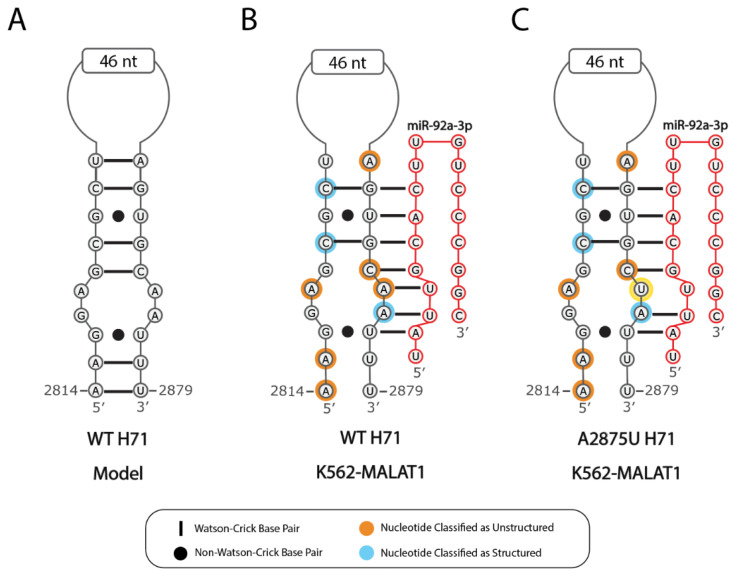
A2875U disrupts the miR-92-3p binding site on H71. (**A**) A schematic is shown for the predicted structure of wild type (WT) H71 in noncancerous cells [15]. Nucleotides with an orange background are classified as unstructured and nucleotides with a blue background are classified as structured. The expected binding of miR-92a-3p (red) to H71 in K562-MALAT1 is shown for (**B**) wild type H71 and (**C**) H71 with the A2875U mutation, which is outlined in yellow. The same disruption to seed binding is expected for miR-363-3p and miR-25-3p (Supplemental Table S1, “Seq-Markup” tab).

Seventeen SNPs have been identified in MALAT1 [54]. rs664589 (C4117G), rs115795653 (A6415G), and rs60151940 (C7151W) are three SNPs that correspond to nucleotides that are predicted to lose structure in K562-MALAT1 (Supplemental Table S1). SNPs in structured RNAs are generally believed to alter the local secondary structure [55], although the severity of alterations can vary and is difficult to predict [56]. Interestingly, SNP rs664589 has been characterized as aiding colorectal cancer progression by inhibiting MALAT1-miR-194-5p binding [57]. Besides SNPs, 655 somatic cancer-associated mutations have been identified in MALAT1 [58]. Fifty-nine mutations (9.0%) correspond to nucleotides predicted to lose structure in K562-MALAT1 (Supplemental Table S1). Such mutations could further weaken the hairpin structures or reduce miRNA binding, particularly if the mutation were to disrupt base pairing in the seed region of the miRNA-binding site. Within H71, the A2875U mutation alters seed-region base pairing for miR-92a-3p, miR-363-3p, and miR-25-3p (Figure 5 and Supplemental Table S1). Although miR-363-3p is associated with tumor suppression in other cancer types [59], miR-92a-3p and miR-25-3p promote progression in cancers like liposarcomas [60]. Moreover, miR-92a-3p was previously found to aid CML by downregulating C/EBPα and subsequently causing cachexia, i.e., severe weight and muscle loss associated with cancer [61]. As predicted previously, this proposed role of H71 in regulating MALAT1-miRNA interactions illustrates how H71 can modulate the different outputs depending on the cellular context [15]. In total, 16 miRNA-binding sites have seed regions within hairpins predicted to become unstructured in K562-MALAT1 (Supplemental Table S1, “SeqMarkup” tab), and may experience reduced binding affinity due to somatic cancer-associated mutations. Additionally, one METTL3/14-binding site, two HuR/ELAV-binding sites, and ten U1-binding sites face similar conditions because of mutations, pointing to complex regulatory pathways that may depend on the K562-MALAT1 structure. Together, the effects of secondary structural loss on miRNA-, U1 snRNA and HuR-, m^6^A-, SNP-, and cancer-associated mutation-related effects in K562 cells represent potential avenues for further characterization of MALAT1 activity in cancer.

### 2.3. PARIS Data Suggest Maintenance of Overall Structure of MALAT1 with Rearrangements of Select Long-Range Interactions

**Figure 6:**
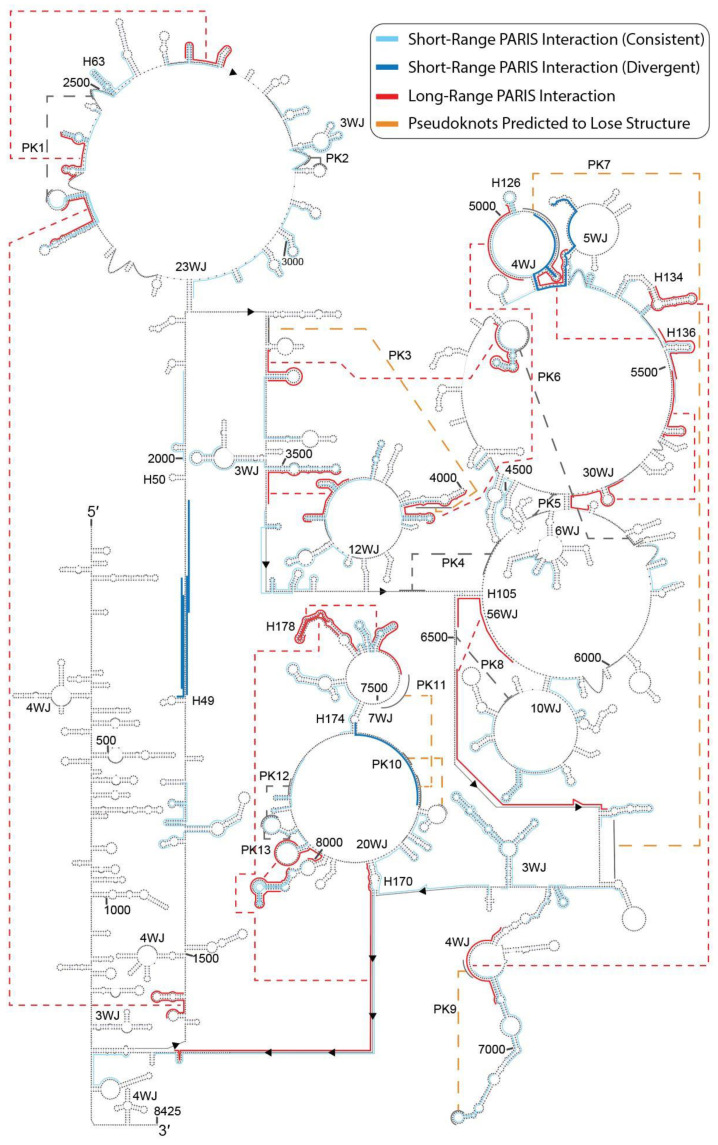
Map of HeLa PARIS data onto the MALAT1 secondary structural model. Short-range (or local) PARIS interactions are depicted with 25 nucleotides flanking the datapoint. Short-range PARIS interactions diverging from the model are dark blue lines, whereas those that are consistent with the consensus model are light blue lines. Long-range PARIS interactions diverging from the model are depicted as red lines and show 50 nucleotides flanking the datapoints. All datapoint coordinates are in Supplemental Table S2. PKs expected-to-be-lost are depicted with dashed orange lines. Labels for secondary structures correspond to those established for the working noncancerous MALAT1 model [15].

PARIS involves sequencing of RNA fragments that were once photocrosslinked-duplexes isolated from psoralen-treated cells [28]. PARIS data for MALAT1 in HeLa cells (herein referred to as HeLa-MALAT1) were compared and mapped to the noncancerous consensus model [15] to see how the MALAT1 secondary structural model is expected to change within the context of HeLa cells (Figure 6, Supplemental Figure S2 and Supplemental Tables S1 and S2) [28,29]. Eighty unique PARIS interactions (Supplemental Table S2) were aligned to the MALAT1 model. Of these, 18 PARIS interactions (22.5%) diverged from hairpins described in the consensus model while 62 local interactions (77.5%) agreed with the hairpins in the consensus model, suggesting that the secondary structure of MALAT1, as it is hypothesized to exist, may be largely maintained in HeLa cells (Figure 6). The 18 PARIS interactions that diverge from the model typically suggest that secondary structural elements undergo structural rearrangement. Of the 18 divergent PARIS interactions, six interactions (33.3%) were denoted as short-range or local interactions and 12 interactions (66.7%) were denoted as long-range interactions, most of which are separated by at least 80 nucleotides in their primary structure in the working noncancerous MALAT1 model (Supplemental Table S2) [15]. The 12 divergent long-range PARIS interactions typically signal the structural rearrangement of multiple hairpins whereas the six short-range PARIS interactions signal the formation of novel structures. Up to 42 hairpins out of 161 hairpins (26.1%) are expected to undergo rearrangement and 119 hairpins (73.9%), which are conserved among mammals [15], appear maintained in the HeLa-MALAT1 model (Supplemental Tables S1 and S2). Thus, with regard to structural alterations of hairpins, rearranging long-range interactions is preferred over novel short-range interactions.

Five of the aforementioned local PARIS interactions occur in predominantly unstructured regions of the working MALAT1 consensus model (Figure 6, dark red lines). Four of these interactions fall between nts 1897 and 1941 (i.e., between H49 and H50) and the fifth interaction falls between nts 7458 and 7461, preceding H174 (Figure 6). The noncancerous HEK293T PARIS data did not highlight any such structures at the corresponding locations (Supplemental Table S2). Together, these five interactions suggest distinct instances of dynamic, novel structures. In contrast, most of the 12 long-range PARIS interactions indicate distinct instances of structural rearrangement (Figure 6, purple lines). Curiously, five of the long-range PARIS interactions and one divergent short-range PARIS interaction start within 561 nucleotides of one another, spanning nts ~4950 to ~5600 (Figure 6 and Supplemental Table S2). This region is largely conserved among mammals as well as some vertebrates [15]. Thus, a core of MALAT1 undergoes structural rearrangement in HeLa cells. The 12 long-range PARIS interactions suggest rearrangement of 39 hairpins, such as H126, H134, H136, and H178. Long-range interactions suggest rearrangement of H105 (coordinates 6446,6564), which notably forms a 56-way junction, and H170 (coordinates 7631,8196), which notably forms a 20-way junction (Figure 6, 56WJ and 20WJ). The PARIS data suggest these long-range interactions are lost in favor of structural rearrangement in HeLa cells, as opposed to general structural loss in K562-MALAT1.

In addition to hairpins, the hypothetical consensus model predicts 13 pseudoknots in noncancerous cells [15]. As previously noted, m^6^A5044 is absent in HeLa cells [15,24,25,26]. This loss of methylation may result in the loss of PK7 as there is a lack of PARIS data for PK7 (coordinates 5038, 6642) in HeLa cells, as previously reported [15]. Instead, PARIS reads (coordinates 5038,5145) suggest formation of a local hairpin [15], as indicated by the sixth divergent short-range PARIS interaction (Figure 6). Additionally, long-range PARIS interactions suggest structural rearrangement of PK3 and PK9 while the structural rearrangement of PK10 and PK11 is supported by short-range interactions. PARIS data do not predict disruption of any other pseudoknots. Unlike hairpins, pseudoknots typically span long ranges of MALAT1 in the working noncancerous model [15]. As a result, loss of many pseudoknots would indicate widespread structural changes in MALAT1. While the loss of these pseudoknots signals some propensity for long-range structural changes, the maintenance of eight pseudoknots reaffirms the trend of structural maintenance within HeLa-MALAT1. Overall, most local secondary structural features are retained in HeLa-MALAT1, with rearrangement of select long-range secondary structures and formation of a small number of novel, local structures.

### 2.4. Predicted Structural Changes in HeLa-MALAT1 Would Impact RNA-Binding Sites and Modifications

Structural rearrangements or novel structures detected in HeLa-MALAT1 means that the structures of RNA- and protein-binding sites underwent changes that may potentially alter their function. MALAT1 has 98 experimentally verified miRNA-binding sites (Supplemental Table S1). Duplex formation in HeLa-MALAT1 suggests disruption of seed region-binding sites for 25 of these validated miRNAs (Supplemental Table S2), which means these binding sites would be less accessible in HeLa cells. The four local PARIS interactions spanning nts 1897–1941 potentially decrease binding site availability for miR-145-5p [62]. miR-145 has been shown to inhibit HeLa cell proliferation by targeting the FSCN1 mRNA transcript [63]. miR-145 is reportedly a tumor suppressor in HeLa cells via the regulation of several proteins, including CDKs and Cyclin D1 [64]. Although miR-145 is downregulated in HeLa cells [65], its function relative to expected changes in the HeLa-MALAT1 structure raise questions regarding the full role of miR-145 in HeLa cells. The remaining PARIS interactions indicate structural rearrangement of binding sites for 24 other supported miRNAs, including miR-200b-3p, miR-20a-5p, and miR-106b-5p [62]. miR-200b is upregulated in cervical cancer and aids cervical cancer metastasis by downregulating FOXG1 [66]. Likewise, miR-20a is upregulated in HeLa cells and leads to the upregulation of the oncogenic protein TNKS2 in HeLa cells [67]. miR-106b is also upregulated in HeLa cells [68] and inhibits HeLa cell proliferation by downregulating PTEN via sponging of miR-106b by the lncRNA PTENP1 [69]. The PARIS data suggest these three latter miRNAs will not be sponged by HeLa-MALAT1, thus possibly aiding HeLa cell growth and survival. Collectively, these studies suggest the presence of complex miRNA-lncRNA-mRNA networks that may be disrupted by changes to MALAT1 secondary structure in HeLa cells. Additional work is required to elucidate the full pathways governed by such miRNAs and to fully understand how structural changes in MALAT1 affect miRNA function in HeLa cells.

Besides miRNAs, MALAT1 has been described as forming intermolecular RNA–RNA interactions with rRNA and U1 snRNA [70,71]. The structural status of one of the five rRNA-binding sites (C2700) and two of the ten U1 snRNA-binding sites (nts 1825–1925 and 6985–7045) is changed in HeLa-MALAT1 (Supplemental Table S1). Because few sites are affected, little to no significant alteration to MALAT1-mediated U1 snRNA and rRNA function is expected in HeLa cells. Additionally, protein-binding sites on MALAT1 are expected to become less available as a result of structural rearrangement throughout HeLa-MALAT1. Three METTL3/14-binding sites (nts 2412–2416, 5042–5046 and 8179–8184) and one HuR/ELAV1-binding site (nts 3248–3258) are hypothesized to undergo structural rearrangement, as indicated by the PARIS data. As previously described, the lack of affinity for ssRNA or dsRNA makes analysis of novel METTL3/14 function with regard to MALAT1 difficult [42]. However, the METTL3/14 complex shows some increased affinity for single-stranded nucleic acids [72], so aberrant m^6^A levels are possible under such circumstances as a result of MALAT1 rearrangement. Unlike the K562 cells, loss of HuR-MALAT1 binding is expected in HeLa cells as structural rearrangement will make the HuR-binding site less available. This loss mirrors the aforementioned functions of HuR-MALAT1 binding in breast cancer [36], suggesting HuR-MALAT1 binding may be decreased in HeLa cells in order to target CD133 expression and subsequently promote cancer progression. Because only one HuR-binding site is expected to undergo structural rearrangement, the repercussions on HuR function may be muted. Although probing of this particular pathway is needed to confirm such a hypothesis, a possible role of HuR-MALAT1 binding is more compelling in HeLa cells than in K562 cells.

Interestingly, several RNA modification sites identified in MALAT1 isolated from HeLa cells occur in structurally rearranged regions: 19 m^6^A sites, five m^5^C sites (C4834, C5518, C5520, C5538, and C5539), and one Am (2′-O-methyladenosine) modification site (A1909) (Supplemental Tables S1 and S2). m^5^C modifications have been found to regulate chromatin-related roles in other lncRNAs, such as HOTAIR and Xist, for this modification often occurs specifically in regions of the lncRNA that interact with chromatin-associated protein complexes [73]. The five aforementioned m^5^C sites were specifically identified in HeLa cells (see Supplemental Table S1). All five m^5^C sites in MALAT1 are clustered within 705 nucleotides of each other, with four of them clustered within 21 nucleotides (Supplemental Table S1). Thus, because MALAT1 binds active chromatin, a novel structure in HeLa-MALAT1 may promote a distinct and cancer-specific chromatin-associated complex via m^5^C [74]. Moreover, the existence of modified nucleotides in MALAT1 and the diversity of RNA modifications, along with advances in modification detection, may result in the discovery of novel MALAT1 modifications that can be implemented as biomarkers [75]. Thus, integrating PARIS and RNA modification data yielded insights into how RNA modifications, particularly m^5^C, may influence MALAT1 function in HeLa cells.

MALAT1 has 17 SNPs [54]. The HeLa-MALAT1 data suggest structural rearrangements for three SNP sites: rs11540782 (U1876C), rs1056816 (A4872K), and rs79910129 (G3247W) (Supplemental Table S1). As previously stated, the exact effects of a given SNP on secondary structure can vary but often result in the disruption of duplexes and loss of secondary structure [56]. As such, based on the PARIS data, no major cellular changes are expected in HeLa cells related to the MALAT1 SNPs. Of the 655 somatic cancer-associated mutations that have been identified in MALAT1 [58], 102 mutations (15.6%) occur in regions predicted to undergo structural rearrangement in HeLa-MALAT1. Mutations within PARIS interactions are liable to destabilize the corresponding RNA duplexes but are also likely to disrupt the binding sites, thus decreasing binding of molecules like miRNAs. Eight miRNA seed-region binding sites in HeLa-specific PARIS interactions are altered by mutations, as are one METTL3/14-binding site and one HuR/ELAV1-binding site. A U5520 insertion alters the seed-region binding sites of three miRNAs within a long-range PARIS interaction (coordinates 5503, 5708): miR-17-5p, miR-20ab-5p, and miR-106b-5p. As discussed previously, free miR-20a is expected to aid HeLa cells via TNKS2 expression [67]. Both miR-17-5p and miR-106b-5p are described as oncogenic in cervical cancer [76,77]. miR-17-5p targets TGFBR2 and stimulates proliferation, and miR-106b-5p promotes PTEN downregulation to achieve similar effects [69]. Hence, there is the potential for somatic cancer-associated mutations to regulate MALAT1 function through structural changes in HeLa cells.

## 3. Materials and Methods

### 3.1. Dataset Acquisition

All sequence datasets used in this analysis were accessed using the Gene Expression Omnibus. DMS-Seq data from K562 cells were extracted from data file GSM1297493-GSM1297494 (GSE45803) [27]. PARIS data from HeLa cells were extracted from data file GSM1917754 (GSE74353), representing high RNase data [28,29]. K562 DMS-Seq and HeLa PARIS data were mapped to human MALAT1 relative to hg38 using the open source platform Galaxy [78] and the UCSC Genome Browser [79]. VARNA was used to visualize the MALAT1 secondary structure [80]. Human MALAT1 nucleotide positions correspond to accession NR_002819.2 and ENST00000534336.1.

The data for miRNA-binding sites, U1 snRNA-binding sites, rRNA-binding sites, protein-binding sites, RNA modifications, SNPs, and somatic mutations associated with cancer were previously curated by McCown et al. [15] (Supplemental Table S1) and updated as follows: miRNA data were downloaded from ENCORI (Sun Yat-sen University, Guangzhou, China) on 19 August 2020 [81]. Only experimentally verified miRNAs were considered in our analysis. Additional validated U1 snRNA sites were reported by Cai et al. [82]. Additional m^6^A modification data were downloaded from m^6^AVAR (Sun Yat-sen University, Guangzhou, China) and are accurate as of 1 November 2020 [26]. MALAT1 somatic mutations associated with cancer were accessed from the National Cancer Institute Genomic Data Commons (Bethesda, MD, USA) and were accurate as of 1 April 2020 [58].

### 3.2. Dataset Analysis and Comparison to MALAT1 Secondary Structural Model

K562 DMS-Seq data pertaining to MALAT1 were extracted and examined with respect to the previously determined hypothetical MALAT1 secondary structural model in noncancerous human foreskin fibroblasts [15]. As the ensuing analysis sought to investigate the hypothetical differences in structure as opposed to establish a new model or identify concrete structural changes, no data were excluded on the basis of ambiguity with respect to the noncancerous model [15]. Thresholds for determining whether DMS-Seq data indicated structure were established using several verified hairpins: H190 (triple helix-containing hairpin) [15] and H191- H194 (mascRNA) (Figure 2) [14,83]. Because MALAT1 expression is higher in cancer cells, including leukemia, than in noncancerous tissue [84], the threshold value of structure calls for DMS-Seq data was set at 250 reads rather than 20 [15]. This threshold of 250 reads was established in a manner similar to the threshold of 20 determined for the consensus MALAT1 structure [15]. Briefly, DMS-Seq labels only adenosine and cytidine in a statistically significant manner; therefore, only these residues were considered in our structural analysis. Adenosine and cytidine residues having no more than 250 DMS-Seq reads were classified as structured. Adenosine and cytidine residues with more than 250 DMS-Seq reads were considered unstructured. We chose 250 as the threshold because this value was approximately midway between nucleotides that were known to be unstructured (C8291 and A8292 at 314 and 322 reads, respectively) in H190 and nucleotides known to be structured (e.g., m^1^A8398 at 217 reads) in H190-H194 (Supplemental Table S1) [14,83,85].

Like the noncancerous fibroblast DMS-Seq data, K562 DMS-Seq data did not contain nts 1–1280 of MALAT1 because the major isoform of MALAT1 is nts 1281–8425 [83] nor nts 4258–5838 (see Supplemental Table S1). Of the 194 hairpins predicted by McCown et al., 124 hairpins were predicted between nts 1281–4258 and nts 5838–8425 [15]. Of these, 23 hairpins were predicted using PARIS data and were thus excluded from the analysis for this cell line. Hence, 101 hairpins were analyzed for structural alterations in K562 cells. Structural alterations within hairpins were determined by comparing the K562 DMS-Seq data to the noncancerous secondary structural model of human MALAT1 excluding PARIS-derived hairpins, as well as to human foreskin fibroblasts [27,29]. As all 13 pseudoknots were established using PARIS data, structural changes in pseudoknots were not considered in the K562 MALAT1 model.

To verify the hypothesis that the unstructured adenosines and cytidines have higher DMS reactivity values than structured adenosines and cytidines (Supplemental Table S1, see U Test of Noncancerous Data tab and U Test of K562-MALAT1 Data tab), we conducted a Mann–Whitney U test on the DMS-Seq data for the structured and unstructured adenosines and cytidines in noncancerous MALAT1 [15] and K562-MALAT1. The DMS-Seq values from all adenosines and cytidines classified as structured or unstructured were tabulated. For the noncancerous MALAT1 model, DMS-Seq data derived from fibroblasts [27] were sorted into structured and nonstructured categories based on their presence in structured or unstructured regions of the consensus noncancerous MALAT1 model. These DMS-Seq values were then ordered from smallest to largest, ranked, and subjected to a U test. This U test produced a statistically significant result, with a *p* value of ~0. For the K562-MALAT1 U test, structured nucleotides were all adenosine/cytidine nucleotides in stems of hairpins that maintained more than 50% of the base pairs (Supplemental Table S1, see Hairpin Coordinates tab). PARIS-derived hairpins were included. Ranks for structured and unstructured DMS-Seq values were determined and subjected to a U test. The worse performing *U*, corresponding to the unstructured portion of the K562-MALAT1 model, was subjected to a *z* statistic calculation and was determined to be significant with a *p* value of ~0, demonstrating that the mean values of structured DMS-Seq adenosines and cytidines do not differ randomly from the mean values of the unstructured DMS-Seq adenosines and cytidines and, as stated, the null hypothesis can be rejected. Although these U tests suggest that the unstructured DMS-Seq values are larger than the structured DMS-Seq values, please bear in mind that these DMS-Seq values are not truly independent observations due to nearest-neighbor effects of nucleotides within helices. Two additional U tests were also conducted to compare the DMS-Seq values for adenosine/cytidine nucleotides classified as unstructured in cancerous K562 and noncancerous fibroblasts cells and structured in cancerous and noncancerous cells. The DMS-Seq values for all structured and all unstructured nucleotides in both cell types were sorted into respective columns, ranked, and subjected to U tests. Both U tests produced statistically significant results, with *p* values of ~0. Please note that these statistical tests may be impacted by the different thresholds applied to the noncancerous and cancerous DMS-Seq data, thereby ensuring different means.

HeLa PARIS data were examined with respect to a previously determined hypothetical MALAT1 secondary structural model in noncancerous HEK293T cells [15]. Because the HEK293T PARIS samples were prepared under high RNase conditions, we likewise examined the high RNase dataset for HeLa cells [28,29]. First, we removed low quality RNA-seq reads, which were lower than Q30 on the Illumina quality score metrics for each read. Reads that formed duplexes outside the MALAT1 coordinates were excluded. Next, PCR duplicates were removed from our analysis by barcode matching in Linux. Finally, PARIS reads that overlapped by 20 or fewer nts were compressed into one region of at least 10 nts and defined as a double-stranded region for any region having at least three PARIS reads. Importantly, PARIS data from HEK293T and HeLa cells were not available for nts 1–1280 of MALAT1 because the major isoform of MALAT1 is nts 1281–8425 [83]; therefore, nts 1–1280 of MALAT1 were not examined for structural alterations in HeLa cells. Structural alterations within hairpins and pseudoknots were determined by comparing the HeLa PARIS data to the noncancerous secondary structural model of human MALAT1, as well as to HEK293T PARIS data [28,29]. When aligning HeLa PARIS data to MALAT1, the PARIS data were assigned specific ranges of nts (see Supplemental Table S2), occasionally creating an apparent overlap among the PARIS interactions (see Figure 6). Because these overlaps are likely artificial, they were not considered to be indicating dynamic structures but to be indicating PARIS interactions in close proximity.

### 3.3. Data and Software Availability

All data and software are freely available at their designated repositories as indicated above. To the best of our knowledge, there are no restrictions or embargoes in place on any of these data.

## 4. Conclusions

Analyzing the K562-MALAT1 and HeLa-MALAT1 models provides insights into the roles and mechanisms of MALAT1 in two different cancer cell lines: K562 and HeLa cells. Based on the putative structural changes with respect to a working model in noncancerous cells, we hypothesize that MALAT1 possesses different secondary structures in both K562 and HeLa cells. However, the nature of these structural changes is distinct to each cancer type and does not appear to have obvious overlaps. In K562 cells, we predict that at least 30% of all MALAT1 hairpins will lose at least 50% of their base pairs (Figure 1, Figure 2 and Figure 3). This putative loss of structure may increase the propensity for miRNA sponging by K562-MALAT1, causing profound effects on cancer cell function. In contrast, PARIS data in HeLa cells suggest that long-range interactions occur in HeLa-MALAT1 that are not seen in noncancerous cells, likely due to alternative structuring of some hairpins and select pseudoknots in MALAT1, although most local secondary structures are preserved. The novel long-range interactions that we predict in HeLa-MALAT1 are expected to decrease instances of miRNA sponging and perhaps alter the functional readout of m^5^C methylations on MALAT1, having widespread consequences in K562 cells. Toggling of structures near m^5^C marks may influence the chromatin restructuring and gene expression in HeLa cells. Whereas K562-MALAT1 is predicted to have increased miRNA-sponging capabilities in K562 cells, alterations to m^5^C-containing structures in HeLa-MALAT1 may point to novel MALAT1-chromatin interactions, hinting at diverse biological processes being coordinated by MALAT1. The full extent to which the proposed structural alterations affect cancer cell development and progression await experimental validation, from establishing a secondary structural model of MALAT1 to confirming isolated structure-function relationships. However, we have identified the possible structural differences between MALAT1 in different cellular contexts that could exacerbate K562 and HeLa cells, leading to mechanistic insights regarding the complex cancer-specific functions of MALAT1.

## Data Availability

The data that support the findings of this study are openly available at the Gene Expression Omnibus at http://www.ncbi.nlm.nih.gov/geo using GSE or GSM numbers as indicated within the article and/or its Supplementary Materials.

## Supplementary Materials

The following are available online at https://www.mdpi.com/2311-553X/7/1/6/s1. Supplemental Figure S1: Poster-size image (approximately 19 in. by 31 in.) of Figure 2 showing K562 DMS-Seq structural changes on the MALAT1 secondary structural model. Supplemental Figure S2: Poster-size image (approximately 19 in. by 31 in.) of Figure 6 showing HeLa PARIS data on the MALAT1 secondary structural model. Supplemental Table S1: Sequence markup and analysis of K562-MALAT1 structural changes on various structure-dependent functions. Supplemental Table S2: Analysis of HeLa-MALAT1 structural changes on various structure-dependent functions.
