# Supplementary material for: Secondary Structural Model of MALAT1 Becomes Unstructured in Chronic Myeloid Leukemia and Undergoes Structural Rearrangement in Cervical Cancer
Source: Noncoding RNA. 2021 Jan 13;7(1):6. doi: 10.3390/ncrna7010006 (PMC7838788; doi:10.3390/ncrna7010006)
Supplement: Supplementary file 1 [file 210112_Supplemental Information.pdf › Supplementary_Files_Rebuttal_B/210112_Supplemental Information.pdf]

Supplementary Information

Secondary Structural Model of MALAT1 Becomes Unstructured in Chronic Myeloid Leukemia and Undergoes Structural Rearrangement in Cervical Cancer

Matthew C. Wang, Phillip J. McCown, Grace E. Schiefelbein and Jessica A. Brown

Supplemental Figures

Supplemental Figure S1. Poster-size image (approximately 19 in. by 31 in.) of Figure 2 showing K562 DMS-Seq structural changes on the MALAT1 secondary structural model.

Supplemental Figure S2. Poster-size image (approximately 19 in. by 31 in.) of Figure 6 showing HeLa PARIS data on the MALAT1 secondary structural model.

Supplemental Tables

Supplemental Table S1. Sequence markup and analysis of K562-MALAT1 structural changes on various structure-dependent functions.

Supplemental Table S2. Analysis of HeLa-MALAT1 structural changes on various structure-dependent functions.

Supplemental Figure Legends

Supplemental Figure S1: Poster-size image (approximately 19 in. by 31 in.) of Figure 2 showing K562 DMS-Seq structural changes on the MALAT1 secondary structural model. K562 DMS-Seq data were available for nts 1284–4257 and nts 5839–8425. Of those indicating change in structure relative to the noncancerous consensus model, adenosine and cytidine residues that are predicted to lose structure are depicted in orange (733 datapoints), and those residues that are predicted to gain structure are depicted in blue (317 datapoints). Orange marks in hairpins have been extended to cover both nucleotides in a given base pair, and base pairs marked as unstructured have the two nucleotides moved apart to emphasize loss of structure. Labels for secondary structures correspond to those established for the working MALAT1 model in noncancerous cells [1]. Please note that PARIS-derived hairpins appear in the consensus model, although these hairpins were not considered in the differential structural analysis of K562-MALAT1.

Supplemental Figure S2: Poster-size image (approximately 19 in. by 31 in.) of Figure 6 showing HeLa PARIS data on the MALAT1 secondary structural model. Short-range (or local) PARIS interactions are depicted with 25 nucleotides flanking the datapoint. Short-range PARIS interactions diverging from the model are dark blue lines, whereas those that are consistent with the consensus model are light blue lines. Long-range PARIS interactions diverging from the model are depicted as red lines and show 50 nucleotides flanking the datapoints. All datapoint coordinates are in Supplemental Table S2. PKs expected to be lost are depicted with dashed orange lines. Labels for secondary structures correspond to those established for the working noncancerous MALAT1 model [1].

Supplemental Table Descriptions

Supplemental Table S1

The tab labeled “Abbreviations” alphabetically lists all abbreviations used in this article.

The tab labeled “SeqMarkup” contains the MALAT1 nucleotide sequence (Row 1), nucleotide coordinates (Row 2), noncancerous structural data (Rows 3–6), and the structural consensus sequence previously assembled (Row 7) [1]. Cells colored blue represent structured nucleotides, whereas cells colored orange represent unstructured nucleotides. The “SeqMarkup” tab also contains the K562 DMS-Seq data [27] (Row 11) as well as an analysis of which nucleotides register as more structured (Row 12), less structured (Row 13), or unchanged in structure (Row 14) in K562 cells based on DMS-Seq data compared to the MALAT1 noncancerous consensus sequence in Row 7. A result of 1 indicates an affirmative response, and a result of 0 indicates a negative response (e.g., a nucleotide with a value of 1 in Row 12 is predicted to gain structure relative to the corresponding nucleotide in the noncancerous consensus model (Row 7), whereas a nucleotide with a value of 1 in Row 13 is predicted to lose structure relative to the corresponding nucleotide in the noncancerous consensus model). The HeLa PARIS data [28,29] is represented in Row 15, where a value of 1 indicates a relevant PARIS interaction at that location (see Supplemental Table S2 for list of PARIS interactions). The hairpins predicted previously [1] (Row 18), validated miRNA-binding sites, U1/rRNA/RPS6-binding sites, protein-binding sites, modified nucleotides, SNPs, and somatic cancer-associated mutations (Rows 19–26, respectively) are aligned to the MALAT1 nucleotide sequence. Hairpins use “>” to denote the 5’ side and “<” to denote the 3’ side. Somatic cancer-associated mutation uses “>” to indicate base substitution, “ins” to indicate insertions, and “del” to indicate deletions.

The tab labeled “U Test of Noncancerous Data” lists the values and shows calculations used to perform the Mann-Whitney U test on noncancerous DMS-Seq data [1]. The column labeled “StructuredReads” is a listing of all the DMS-Seq values for adenosines and cytidines that were classified as structured in the original noncancerous model [1]. The column labeled “UnstructuredReads” is a listing of all the DMS-Seq values for adenosines and cytidines that were classified as unstructured in the original noncancerous model [1]. The column labeled “StructuredRanks” contains the ranks for each of the DMS-Seq reads found under the “StructuredReads” column. The column labeled “UnstructuredRanks” contains the ranks for each of the DMS-Seq reads found under the “UnstructuredReads” column. Column G contains parameters for all outputs used in calculating the U, z statistic, and p value from the Mann-Whitney U test. Column H contains the formula and numerical results for *R_Structured*, *R_Unstructured*, *N_Structured*, *N_Unstructured*, *U_Structured*, *U_Unstructured*, the z statistic, and the p value.

The tab labeled “U Test of K562-MALAT1 Data” lists the values and shows calculations used to perform the Mann-Whitney U test on K562 DMS-Seq data. The column labeled “StructuredReads” is a listing of all the DMS-Seq values for adenosines and cytidines that were classified as structured in our K562-MALAT1 model. The column labeled “UnstructuredReads” is a listing of all the DMS-Seq values for adenosines and cytidines that were classified as unstructured in our K562-MALAT1 model. The column labeled “StructuredRanks” contains the ranks for each of the DMS-Seq reads found under the “StructuredReads” column. The column

labeled “UnstructuredRanks” contains the ranks for each of the DMS-Seq reads found under the “UnstructuredReads” column. Column G contains parameters for all outputs used in calculating the *U*, *z* statistic, and *p* value from the Mann-Whitney U test. Column H contains the formula and numerical results for *R_Structured*, *R_Unstructured*, *N_Structured*, *N_Unstructured*, *U_Structured*, *U_Unstructured*, the *z* statistic, and the *p* value.

The tab labeled “U Test of Unstructured Data” lists the values and shows calculations used to perform the Mann-Whitney U test on DMS-Seq data classified as unstructured. The column labeled “UnstructuredReads Noncancerous” is a listing of all the DMS-Seq values for adenosines and cytidines that were classified as unstructured in the original noncancerous model [1]. The column labeled “UnstructuredReads Cancerous” is a listing of all the DMS-Seq values for adenosines and cytidines that were classified as unstructured in our K562-MALAT1 model. The column labeled “UnstructuredRanks Noncancerous” contains the ranks for each of the DMS-Seq reads found under the “UnstructuredReads Noncancerous” column. The column labeled “UnstructuredRanks Cancerous” contains the ranks for each of the DMS-Seq reads found under the “UnstructuredReads Cancerous” column. Column G contains parameters for all outputs used in calculating the *U*, *z* statistic, and *p* value from the Mann–Whitney U test. Column H contains the formula and numerical results for *R_Noncancerous*, *R_Cancerous*, *N_Noncancerous*, *N_Cancerous*, *U_Noncancerous*, *U_Cancerous*, the *z* statistic, and the *p* value.

The tab labeled “U Test of Structured Data” lists the values and shows calculations used to perform the Mann–Whitney U test on DMS-Seq data classified as structured. The column labeled “StructuredReads Cancerous” is a listing of all the DMS-Seq values for adenosines and cytidines that were classified as structured in our K562-MALAT1 model. The column labeled “StructuredReads Noncancerous” is a listing of all the DMS-Seq values for adenosines and cytidines that were classified as unstructured in the original noncancerous model [1]. The column labeled “StructuredRanks Cancerous” contains the ranks for each of the DMS-Seq reads found under the “StructuredReads Cancerous” column. The column labeled “StructuredRanks Noncancerous” contains the ranks for each of the DMS-Seq reads found under the “StructuredReads Noncancerous” column. Column G contains parameters for all outputs used in calculating the *U*, *z* statistic, and *p* value from the Mann–Whitney U test. Column H contains the formula and numerical results for *R_Cancerous*, *R_Noncancerous*, *N_Cancerous*, *N_Noncancerous*, *U_Cancerous*, *U_Noncancerous*, the *z* statistic, and the *p* value.

The tab labeled “Hairpin Coordinates” gives the hairpins previously determined [1] and their coordinates, as well as conservation data and their presence in K562-MALAT1 and HeLa-MALAT1. Highlighted hairpins were originally determined [1] solely using noncancerous HEK293T PARIS data.

The tab labeled “Structured Loops” lists the structured loops previously determined [1], their respective hairpins, and their presence in K562-MALAT1 or HeLa-MALAT1.

The tab labeled “miRNAs” lists the examined miRNAs, their binding positions and the associated MALAT1 nucleotide sequence, and whether their binding sites are altered by predicted structural changes in K562 and HeLa cells.

The tab labeled “U1-rRNA-RPS6” lists the examined U1-, rRNA, and RPS6-binding sites, their positions on MALAT1, conservation data, and whether their binding sites are altered by predicted structural changes in K562-MALAT1 and HeLa-MALAT1.

The tab labeled “Proteins” lists the examined protein-binding sites, their positions on MALAT1, conservation data, and whether their binding sites are altered by predicted structural changes in K562 and HeLa cells. The preference of the associated proteins for single-stranded RNA or double-stranded RNA is also noted.

The tab labeled “RNA Modifications” lists the examined RNA modification sites, their positions on MALAT1, method and its corresponding reference, cell type and whether their sites are altered by predicted structural changes in K562-MALAT1 and HeLa-MALAT1.

The tab labeled “SNPs” lists the examined SNP sites, their positions on MALAT1, and whether their sites are altered by predicted structural changes in K562-MALAT1 and HeLa-MALAT1.

The tab labeled “Cancer Mutations” lists the examined cancer-associated mutation sites, their positions on MALAT1, the type of mutation, number of cases, and whether the sites are altered by predicted structural changes in K562-MALAT1 and HeLa-MALAT1. Highlighted mutations fall outside of range (nts 1–8425) of MALAT1 examined in this work.

Supplemental Table S2

The tab labeled “HeLa PARIS data” lists the 80 examined PARIS interactions and their coordinates. The 18 PARIS interactions that diverge from the noncancerous structural model [1] are highlighted in yellow. For each PARIS interaction, its coordinates, the surrounding MALAT1 nucleotides, and its classification as short-range or long-range is given. Highlighted PARIS interactions also list impacted hairpins, miRNA seed-region binding sites, U1- and rRNA-binding sites, protein-binding sites, RNA modification sites, SNP sites, and number of cancer-associated mutation sites.

The tab labeled “HEK293T PARIS data” lists the 95 MALAT1 PARIS interactions previously investigated in HEK293T cells and their coordinates [1].

Supplemental Reference

1. McCown, P. J., Wang, M. C., Jaeger, L. & Brown, J. A. Secondary structural model of human MALAT1 reveals multiple structure–function relationships. *Int. J. Mol. Sci.* **20**, pii: E5610 (2019).
