# Supplementary material for: Secondary Structural Model of MALAT1 Becomes Unstructured in Chronic Myeloid Leukemia and Undergoes Structural Rearrangement in Cervical Cancer
Source: Noncoding RNA. 2021 Jan 13;7(1):6. doi: 10.3390/ncrna7010006 (PMC7838788; doi:10.3390/ncrna7010006)
Supplement: Supplementary file 1 [file Supplemental Figure 1.pdf › Supplementary_Files_Rebuttal_B/Supplemental Figure 1.pdf]

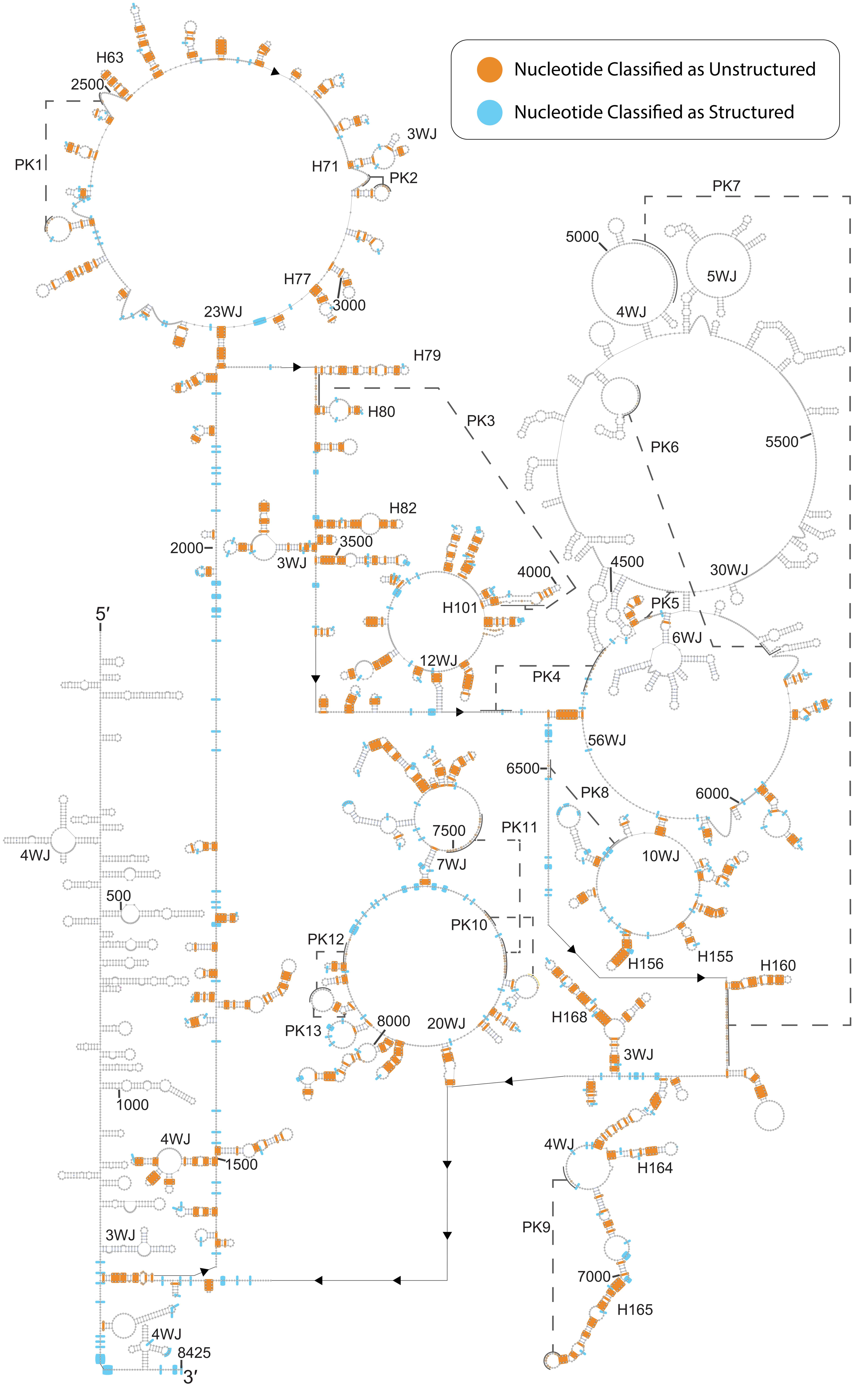

Nucleotide Classified as Unstructured
Nucleotide Classified as Structured
H63
2500
PK1
3WJ
H71
PK2
H77
3000
23WJ
PK7
5000
4WJ
5WJ
H79
H80
PK3
PK6
5500
H82
2000
3500
3WJ
4000
4500
30WJ
H101
PK5
6WJ
12WJ
PK4
5′
56WJ
6500
PK8
6000
4WJ
7500
PK11
7WJ
10WJ
500
PK10
PK12
H156
H155
H160
H168
PK13
8000
20WJ
3WJ
1000
4WJ
H164
4WJ
1500
PK9
3WJ
7000
H165
4WJ
8425
3′
