# Supplementary material for: Secondary Structural Model of MALAT1 Becomes Unstructured in Chronic Myeloid Leukemia and Undergoes Structural Rearrangement in Cervical Cancer
Source: Noncoding RNA. 2021 Jan 13;7(1):6. doi: 10.3390/ncrna7010006 (PMC7838788; doi:10.3390/ncrna7010006)
Supplement: Supplementary file 1 [file Supplemental Figure 2.pdf › Supplementary_Files_Rebuttal_B/Supplemental Figure 2.pdf]

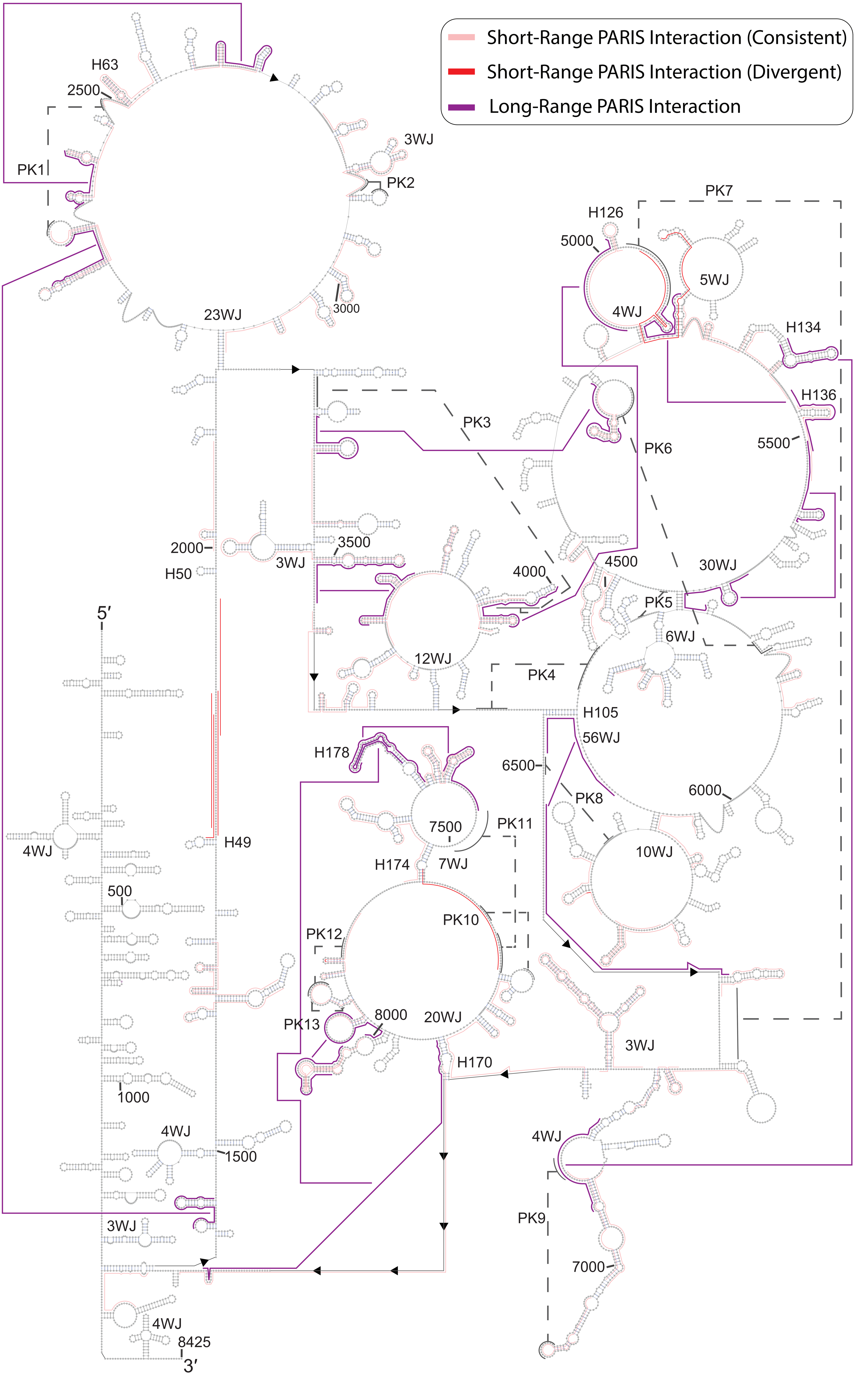

Short-Range PARIS Interaction (Consistent)
Short-Range PARIS Interaction (Divergent)
Long-Range PARIS Interaction
H63
2500
PK1
3WJ
PK2
23WJ
3000
PK7
H126
5000
5WJ
4WJ
H134
H136
5500
PK3
PK6
2000
H50
3WJ
3500
4000
4500
30WJ
12WJ
PK5
6WJ
PK4
H105
56WJ
5′
H178
6500
PK8
6000
7500
PK11
H49
4WJ
H174
7WJ
10WJ
500
PK10
PK12
PK13
8000
20WJ
3WJ
H170
1000
4WJ
4WJ
1500
PK9
3WJ
7000
4WJ
8425
3′
